# Histological outcomes in HPV-screened elderly women in Denmark

**DOI:** 10.1371/journal.pone.0246902

**Published:** 2021-02-11

**Authors:** Gry St-Martin, Petra Hall Viborg, Ane Birgitte Telén Andersen, Berit Andersen, Jette Christensen, Dorthe Ejersbo, Hanne Nørgaard Heje, Kirsten Marie Jochumsen, Tonje Johansen, Lise Grupe Larsen, Elsebeth Lynge, Reza Rafiolsadat Serizawa, Marianne Waldstrøm

**Affiliations:** 1 Center for Epidemiological research, Nykøbing Falster Hospital, Nykøbing Falster, Denmark; 2 The Danish Clinical Registries (RKKP), Frederiksberg/Aarhus N, Denmark; 3 Department of Public Health Programmes, Randers Regional Hospital, Randers, Denmark; 4 Department of Clinical Medicine, Aarhus University Hospital, Aarhus, Denmark; 5 Department of Pathology, Aalborg University Hospital, Aalborg, Denmark; 6 Department of Pathology, Vejle, Lillebaelt Hospital, Vejle, Denmark; 7 General Practice, Århus C, Denmark; 8 Department of Gynecology and Obstetrics, Odense University Hospital, Odense C, Denmark; 9 Department of Pathology, Randers Regional Hospital, Randers NØ, Denmark; 10 Department of Pathology, Zealand University Hospital, Naestved, Denmark; 11 Department of Public Health, University of Copenhagen, Copenhagen, Denmark; 12 Department of Pathology, Hvidovre Hospital, Hvidovre, Denmark; 13 Institute of Regional Health Science, University of Southern Denmark, Odense, Denmark; Greenebaum Cancer Center, Institute of Human Virology, University of Maryland School of Medicine, UNITED STATES

## Abstract

**Introduction:**

Danish women exit cervical cancer screening at age 65 years, but 23% of cervical cancer cases occur beyond this age. In addition, due to gradual implementation of cervical cancer screening, older women are underscreened by today´s standards. A one-time screening with HPV test was therefore offered to Danish women born before 1948.

**Methods:**

Register based study reporting histology diagnoses and conizations in women found HPV positive in the one-time screening. Number and proportion of women with severe or non-severe histology results were calculated for screened and HPV-positive women by age group or region of residence. Number of women with biopsy and/or conization per case of cervical intraepithelial neoplasia (CIN) grade 2 or worse (CIN2+) or CIN3+ were also calculated by age groups and region.

**Results:**

4,479 (4.1% of screened women) had positive HPV test. 94% of these had one or more additional tests. 2,785 (62%) of HPV-positive women had histology results, and conization was performed in 1,076 (24% of HPV-positive and 1% of all screened women). HPV positivity and CIN3+ detection varied little between regions, but the proportions of HPV positive women undergoing histology varied between regions from 40% to 86% and the proportion with conization from 13% to 36%. Correspondingly, the number of histologies and conizations per CIN3+ detected varied from 5.9 to 11.2 and 1.8 to 4.7, respectively. In total, 514 CIN2+ (0.47% of screened women, 11% of HPV-positive) and 337 CIN3+ (0.31% of screened women, 7.5% of HPV-positive) were diagnosed, including 37 cervical cancer cases.

**Discussion:**

HPV screening of insufficiently screened birth cohorts can potentially prevent morbidity and mortality from cervical cancer but longer follow-up is needed to see if cancer incidence declines in the screened women in the coming years. Management strategies differed among regions which influenced the proportions undergoing biopsy/conization.

## Introduction

Cervical cancer screening has substantially reduced the incidence of cervical cancer [1]. Most countries in Europe recommend cervical screening of women up until the age of 60–65 years [2]. In many high-income countries, however, the cervical cancer incidence in women aged above 65 years is relatively high. This has led to considerations regarding optimal age to stop screening [3,4].

Denmark is no exception: women exit the screening program at age 60–64 years, but 23% of cervical cancer cases in 2011–16 were diagnosed in women aged 65 years or older [5]. This attracted attention and gave rise to debate among clinicians, politicians, and non-governmental organizations on a possible extension of the upper age limit for screening. The Danish cervical screening program was implemented gradually, until all women aged 23 to 59 years were covered in 2006. In 2007, the upper age limit was extended to 64 years, and since 2012, the national screening guidelines have recommended testing for high risk (hr) human papillomavirus (HPV) as an exit-test between age 60 and 64. Women aged below 60 are screened with cytology [6,7].

Realizing that the history of the Danish cervical cancer screening program had left women born before 1948 incompletely screened or unscreened [8], the government decided to offer a one-time HPV screening test to women born before 1948 [9,10]. This initiative was implemented in 2017–18, and 30% of invited women participated of whom 4.1% tested positive for hr-HPV [9]. In this article, we report on histological findings among these HPV-positive women with the aim of contributing to the evidence regarding cervical cancer screening beyond the currently recommended upper age limit.

## Material and methods

The one-time screening initiative for women born before 1948 has been described in detail previously [9]. Briefly, the initiative was part of a wider, national plan for cancer developed by the Ministry of Health, which provided for a single contact to women in the targeted birth cohorts. No ongoing screening or reminders were included in the cancer plan for these cohorts. The initiative was implemented by the five administrative regions of Denmark, following joint planning by the national health authorities and the regions. All women in the targeted birth cohorts received a letter during 2017 from their region of residence with information about the initiative and an invitation to contact their general practitioner (GP) for an HPV screening test or, if needed, for individualized advice about participation. Denmark has a tax-financed, public healthcare system and participation in the screening was voluntary and free of charge to the women. The GP collected a cervical sample which was analyzed for hr-HPV at the public hospital pathology departments. Of 359,763 invited women, 108,585 (30%) were screened, of whom 4,479 (4.1%) were positive for hr-HPV, in the following abbreviated to HPV-positive [9].

Results of the initial HPV tests as well as triage and follow-up tests were recorded in the Danish Pathology Register (DPR), which holds complete cytology and histology results from all pathology departments in Denmark [11]. Test results for the 4,479 women who initially tested HPV-positive were retrieved from 2017 to October 2019 using the civil registration number, which provides a unique identifier for each woman. Data for this study was provided from the Quality Assurance Program of the Danish Regions (RKKP) and the Danish Quality Database for Cervical Cancer Screening (DKLS) aggregated by region or by 5-year groups of birth-year.

Although the initiative was centrally planned, the five regions had some autonomy in designing their screening algorithms, further testing, and management. The Capital region recommended referral of all HPV-positive women for colposcopy. In the four other regions, HPV type 16 and 18 were always recommended referral for colposcopy, and for other hr-HPV-types, cytology was performed on the screening sample and colposcopy recommended if atypical squamous cell of undetermined significance or worse (ASCUS+) was present. Otherwise, repeated HPV testing after 12 months was recommended.

Histology diagnoses in the DPR are coded according to the cervical intraepithelial neoplasia (CIN) classification. Histology was divided into severe diagnoses (CIN2, CIN3, cancer, and unclassifiable CIN) and non-severe diagnosis without indication of treatment (CIN1 and normal histology). Histology diagnoses may stem from biopsies, conizations, or hysterectomies. If more than one histology diagnosis was registered, the most severe was used for the analyses.

The following outcomes are reported by birth-year groups and by region:

- Proportion testing HPV-positive
- Proportion returning for further testing, i.e. followed up
- Proportion having histological examination performed
- Proportion with conization
- Proportion with severe histology (CIN2+ and CIN3+)
- Number of histologies and conizations per detected severe diagnosis (CIN2+ or CIN3+)

95% confidence intervals (CI) for proportions were calculated as Clopper-Pearson exact method. Differences between proportions was tested with two-sample test. Analyses were performed with STATA IC 15.1. This study was approved by the Danish Data Protection Agency via the Central Denmark Region which serves as ethical clearance according to Danish legislation.

## Results

Of 108,585 screened women, 4,479 (4.1%) were HPV-positive, ranging from 4.3% in the youngest screened cohorts to 3.2% in the oldest. Of the 4,479 HPV-positive women, 4,222 (94%) had one or more additional tests performed during the period covered by this study; 2,785 (62% of HPV-positive, 2.5% of all screened women) had one or more histology result registered, 514 (11% of HPV positive, 0.47% of screened) had CIN2+, and 1,076 women (24% of HPV-positive, 1% of all screened women) had conization performed, Table 1.

**Table 1:** Danish women born before 1948 and HPV-screened in 2017–2018.

Screening outcome	Birth year				Total
	1943–1947	1938–1942	1933–1937	1932 or earlier	
**Screened women**	60,921	31,990	12,057	3,617	108,585
**HPV-positive**	2,621	1,322	420	116	4,479
% of screened (95% CI)	4.3% (4.1–4.5)	4.1% (3.9–4.4)	3.5% (3.2–3.8)	3.2% (2.7–3.8)	4.1% (4.0–4.2)
**# with follow-up registered**	2,513	1,246	376	87	4,222
% of HPV-positive (95% CI)	96% (95–97)	94% (93–95)	90% (86–92)	75% (66–83)	94% (94–95)
**Histology performed**^1^	1,661	817	251	56	2,785
% of screened (95% CI)	2.7% (2.6–2.9)	2.6% (2.4–2.7)	2.1% (1.8–2.4)	1.5% (1.2–2.0)	2.6% (2.5–2.7)
% of HPV-positive (95% CI)	63% (61–65)	62% (59–64)	60% (55–64)	48% (39–58)	62% (61–64)
% of women w/follow-up (95% CI)	66% (64–68)	66% (63–68)	67% (62–72)	64% (53–74)	66% (65–67)
**Follow-up but no histology**^2^	852	429	125	31	1,437
% of screened (95% CI)	1.4% (1.3–1.5)	1.3% (1.2–1.5)	1.0% (0.86–1.2)	0.85% (0.58–1.2)	1.3% (1.3–1.4)
% of HPV-positive (95% CI)	33% (31–34)	32% (30–35)	30% (25–34)	27% (19–36)	32% (31–33)
% of women w/follow-up (95% CI)	34% (32–36)	34% (32–37)	33% (28–38)	36% (26–47)	34% (33–35)
**Conization performed**	627	344	85	20	1,076
% of screened (95% CI)	1.0% (0.9–1.1)	1.1% (0.9–1.2)	0.7% (0.6–0.9)	0.6% (0.3–0.9)	1.0% (0.9–1.1)
% of HPV-positive (95% CI)	24% (22–26)	26% (24–28)	20% (16–24)	17% (11–25)	24% (23–25)
% of women w/follow-up (95% CI)	25% (23–27)	28% (25–30)	23% (18–27)	23% (15–33)	25% (24–27)
% of women w/histology (95% CI)	38% (35–40)	42% (39–46)	34% (28–40)	36 (23–50)	39% (37–40)

Numbers and proportions of women screened, HPV-positive, with follow-up, with histology, and with conization by birth-year group.

^1^: From biopsy or conization.

^2^: Follow-up with cytology and/or HPV-test.

Across all cohorts, about two-thirds of women for whom follow-up tests were registered had a cervical histology diagnosis. Due to the lower proportion of HPV-positive women and less follow-up in the older cohorts, the proportion with histology diagnosis varied from 2.7% in the youngest to 1.5% in the oldest cohorts of screened women, and from 63% to 48% of HPV-positive women. A similar pattern was seen for conization. In total, 1.0% of screened women underwent conization varying from 1.0% in the youngest to 0.6% in the oldest cohort, but in each cohort constituting about one-fourth of women with follow-up, Table 1.

**Fig 1:**
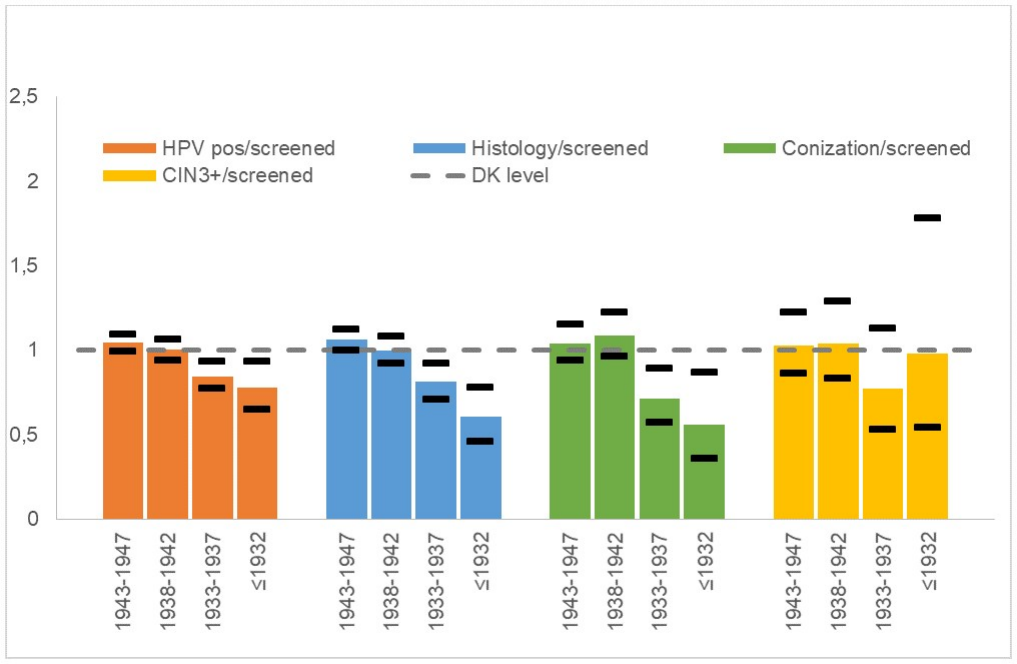
Proportions of women who were HPV positive, had histology performed, had CIN3+, or underwent conization, by birth year, compared to national average.

Overall, CIN3 was diagnosed in 300 women and cervical cancer in 37. These cases constituted 0.31% of screened women and 7.5% of HPV-positive women. All cancer cases occurred in women born after 1937, Table 2. For CIN2+, the proportions were 0.47% of screened women and 11% of HPV-positive women. In total, 3.2 women underwent conization per detected CIN3+ case, and these numbers varied little across cohorts, apart from the oldest group where the numbers were small. For all histology results, including from biopsies, 8.3 women had histology performed for each CIN3+ case diagnosed, Table 2. Fig 1 shows the variation in follow-up screening outcomes by birth year group. Older birth year groups had less follow-up examinations but less variation in CIN3+ detection.

**Table 2:** Histological outcome and proportions with <CIN2, CIN2+ and CIN3+ of screened women, of HPV-positive and of women with follow-up.

Histology outcome	Birth year				Total^2^
	1943–1947	1938–1942	1933–1937	1932 or earlier	
**Women with histology**^1^	1661	817	251	53	2782
No malignancy or dysplasia	1181	552	174	37	1944
CIN 1	163	84	29	5	281
CIN2	60	40	5	#	105
CIN 3/AIS	173	87	29	11	300
CIN unclassified	42	23	5	#	70
Cancer	21	16	0	0	37
Inconclusive or unknown	21	15	9	#	45
**<CIN2**	1344	636	203	42	2225
% of screened (95% CI)	2.2% (2.1–2.3)	2.0% (1.8–2.1)	1.7% (1.5–1.9)	1.2% (0.8–1.6)	2.0% (2.0–2.1)
% of HPV-pos. (95% CI)	51% (49–53)	48% (45–51)	48% (43–53)	36% (27–45)	50% (48–51)
% of women w/histology	81% (79–83)	78% (75–81)	81% (75–86)	75% (62–86)	80% (78–81)
**CIN2+**	296	166	39	13	514
% of screened (95% CI)	0.49% (0.43–0.54)	0.52% (0.44–0.6)	0.32% (0.23–0.44)	0.36% (0.19–0.61)	0.47% (0.43–0.51)
% of HPV-pos. (95% CI)	11% (10–13)	13% (11–14)	9.3% (6.7–12)	11% (6.1–18)	11% (11–12)
% of women w/histology	18% (16–20)	20% (18–23)	16% (11–21)	23% (13–36)	18% (17–20)
**CIN3+**	194	103	29	11	337
% of screened (95% CI)	0.32% (0.28–0.37)	0.32% (0.26–0.39)	0.24% (0.16–0.35)	0.30% (0.15–0.54)	0.31% (0.28–0.35)
% of HPV-pos.(95% CI)	7.4% (6.4–8.5)	7.8% (6.4–9.4)	6.9% (4.7–9.8)	9.5% (4.8–16)	7.5% (6.8–8.3)
% of women w/histology	12% (10–13)	13% (10–15)	12% (7.9–16)	20% (10–32)	12% (11–13)
**Numbers of procedures per diagnosis**					
Conizations per CIN2+ case (95% CI)	2.1 (1.8–2.4)	2.1 (1.8–2.5)	2.2 (1.5–3.2)	1.5 (0.8–3.1)	2.1 (1.9–2.3)
Conizations per CIN3+ case (95% CI)	3.2 (2.8–3.8)	3.3 (2.7–4.2)	2.9 (1.9–4.5)	1.8 (0.9–3.8)	3.2 (2.8–3.6)
Histology performed per CIN2+ case	5.6 (5.0–6.3)	4.9 (4.2–5.8)	6.4 (4.6–9.0)	4.3 (2.4–7.9)	5.4 (4.9–6.0)
Histology performed per CIN3+ case	8.6 (7.4–9.9)	7.9 (6.5–9.7)	8.7 (5.9–12.7)	5.1 (2.7–9.7)	8.3 (7.4–9.3)

Conizations per CIN2+ or CIN3+ detected case by birth-year group. For women with more than one histology diagnosis, the most severe is used.

^1^: From biopsy or conization.

^2^: Cells with fewer than 3 women are excluded for reasons of privacy (marked #). Totals may thus differ between tables.

The proportion of HPV-positive women varied from 3.4% of screened women in Southern Denmark to 4.8% in Central Denmark (p < 0.0001), with the other regions in between, Table 3. The follow-up proportions were high in all regions, but type of follow-up varied considerably. Follow-up with histology varied from 1.8% of screened women in Southern Denmark to 3.5% in the Capital Region (p < 0.0001). In the Capital Region, 90% of women with follow-up had histology performed, 42% in Central Denmark Region, with the other regions in between. Conizations varied from 0.5% of screened women in Southern Denmark to 1.9% in Northern Denmark (p < 0.0001). In total, 0.31% of screened women had CIN3+ detected, Table 4.

**Table 3:** Numbers and proportions of women screened, HPV positive, with follow-up, with histology, and with conization by region.

Screening outcome	Region^1^					Total
	Capital	Zealand	South	Central	North	
**Screened women**	**30,498**	**17,807**	**25,212**	**23,654**	**11,414**	**108,585**
**HPV-positive**	1,246	736	855	1,130	512	4,479
% of screened (95% CI)	4.1% (3.9–4.3)	4.1% (3.8–4.4)	3.4% (3.2–3.6	4.8% (4.5–5.0)	4.5% (4.1–4.9)	4.1% (4.0–4.2)
**# with follow-up test(s)**	1,193	688	789	1,078	474	4,222
% of HPV positive (95% CI)	96% (94–97)	93% (91–95)	92% (90–94)	95% (94–97)	93% (90–95)	94% (94–95)
**Histology performed**^2^	1,073	488	451	453	320	2,785
% of screened (95% CI)	3.5% (3.3–3.7)	2.7% (2.5–3.0)	1.8% (1.6–2.0)	1.9% (1.7–2.1)	2.8% (2.5–3.1)	2.6% (2.5–2.7)
% of HPV positive (95% CI)	86% (84–88)	66% (63–70)	53% (49–56)	40% (37–43)	63% (58–67)	62% (61–64)
% of women w/follow-up (95% CI)	90% (88–92)	71% (67–74)	57% (54–61)	42% (39–45)	68% (63–72)	66% (65–67)
**Follow-up but no histology**^3^	120	200	338	625	154	1,437
% of screened (95% CI)	0.39% (0.33–0.47)	1.1% (0.97–1.3)	1.3% (1.2–1.5)	2.6% (2.4–2.9)	1.3% (1.1–1.6)	1.3% (1.3–1.4)
% of HPV-positive (95% CI)	9.6% (8.0–11)	27% (24–31)	40% (36–43)	55% (52–58)	30% (26–34)	32% (31–33)
% of women w/follow-up (95% CI)	10% (8.4–12)	29% (26–33)	43% (39–46)	58% (55–61)	32% (28–37)	34% (33–35)
**Conization performed**	449	130	136	144	217	1,076
% of screened (95% CI)	1.5% (13–16)	0.73% (0.61–0.87)	0.54% (0.45–0.64)	0.6% (0.51–0.71)	1.9% (1.7–2.2)	1.0% (0.9–1.1)
% of HPV-positive (95% CI)	36% (33–39)	18% (15–21)	16% (14–19)	13% (11–15)	42% (38–47)	24% (23–25)
% of women with follow-up (95% CI)	38% (35–40)	19% (16–22)	17% (15–20)	13% (11–16)	46% (41–50)	25% (24–27)
% of women with histology (95% CI)	42% (39–45)	27% (23–31)	30% (26–35)	32% (28–36)	68% (62–73)	39% (37–40)

^1^: The full names of the Danish regions are Capital Region, Region Zealand, Region of Southern Denmark, Central Denmark Region, North Denmark Region.

^2^: From biopsy or conization.

^3^: Follow-up with cytology and/or HPV-test.

**Table 4:** Histological outcome and proportions with <CIN2, CIN2+ and CIN3+ of screened women, of HPV-positive and of women with follow-up. Conizations per CIN2+ or CIN3+ detected case by region.

Histology outcome	Region^1^					Total^3^
	Capital	Zealand	South	Central	North	
**Women with histology**^2^	1,073	488	451	453	319	2,784
Normal histology	789	376	318	273	188	1,944
CIN 1	90	26	32	77	56	281
CIN2	39	24	12	16	15	106
CIN 3/AIS	87	43	63	62	45	300
CIN unclassified	45	6	7	12	#	70
Cancer	9	6	13	6	3	37
Histology inconclusive or unknown	14	7	6	7	12	46
**<CIN2**	879	402	350	350	244	2225
% <CIN2 of screened women (95% CI)	2.9% (2.7–3.1)	2.3% (2.0–2.5)	1.4% (1.2–1.5)	1.5% (1.3–1.6)	2.1% (1.9–2.4)	2.0% (2.0–2.1)
% <CIN2 of HPV positive (95% CI)	71% (68–73)	55% (51–58)	41% (38–44)	31% (28–34)	48% (43–52)	50% (48–51)
% <CIN2 of women with histology (95% CI)	82% (79–84)	82% (79–86)	78% (73–81)	77% (73–81)	76% (71–81)	80% (78–81)
**CIN2+**	180	79	95	96	64	514
% CIN2+ of screened women (95% CI)	0.59% (0.51–0.68)	0.44% (0.35–0.55)	0.38% (0.30–0.46)	0.41% (0.33–0.50)	0.56% (0.43–0.72)	0.47% (0.43–0.51)
% CIN2+ of HPV positive (95% CI)	14% (13–17)	11% (9–13)	11% (9–13)	8% (7–10)	13% (10–16)	11% (11–12)
% CIN2+ of women with histology (95% CI)	17% (15–19)	16% (13–20)	21% (17–25)	21% (17–25)	20% (16–25)	18% (17–20)
**CIN3+**	96	49	76	68	48	337
% CIN3+ of screened women (95% CI)	0.31% (0.26–0.38)	0.28% (0.20–0.36)	0.30% (0.24–0.38)	0.29% (0.22–0.36)	0.42% (0.31–0.58)	0.31% (0.28–0.35)
% CIN3+ of HPV positive (95% CI)	7.7% (6.3–9.3)	6.7% (5.0–8.7)	8.9% (7.1–11)	6.0% (4.7–7.6)	9.4% (7.0–12)	7.5% (6.8–8.3)
% CIN3+ of women with histology (95% CI)	8.9% (7.3–11)	10% (7.5–13)	17% (14–21)	15% (12–19)	15% (11–19)	12% (11–13)
**Numbers of procedures per diagnosis**						
Conizations per CIN2+ case (95% CI)	2.5 (2.1–3.0)	1.6 (1.2–2.2)	1.4 (1.1–1.9)	1.5 (1.2–1.9)	3.4 (2.6–4.5)	2.1 (1.9–2.3)
Conizations per CIN3+ case (95% CI)	4.7 (3.8–5.8)	2.7 (1.9–3.7)	1.8 (1.4–2.4)	2.1 (1.6–2.9)	4.5 (3.3–6.2)	3.2 (2.8–3.6)
Histology performed per CIN2+ case (95% CI)	6.0 (5.1–7.0)	6.2 (4.9–7.8)	4.7 (3.8–5.9)	4.7 (3.8–5.9)	5.0 (3.8–6.5)	5.4 (4.9–6.0)
Histology performed per CIN3+ case (95% CI)	11.2 (9.1–13.8)	10.0 (7.4–13.3)	5.9 (4.7–7.6)	6.7 (5.2–8.6)	6.7 (4.9–9.0)	8.3 (7.4–9.3)

^1^: The full names of the Danish regions are Capital Region, Region Zealand, Region of Southern Denmark, Central Denmark Region, North Denmark Region.

^2^: From biopsy or conization.

^3^: Cells with fewer than 3 women are excluded for reasons of privacy (marked #). Totals may thus differ between tables.

**Fig 2:**
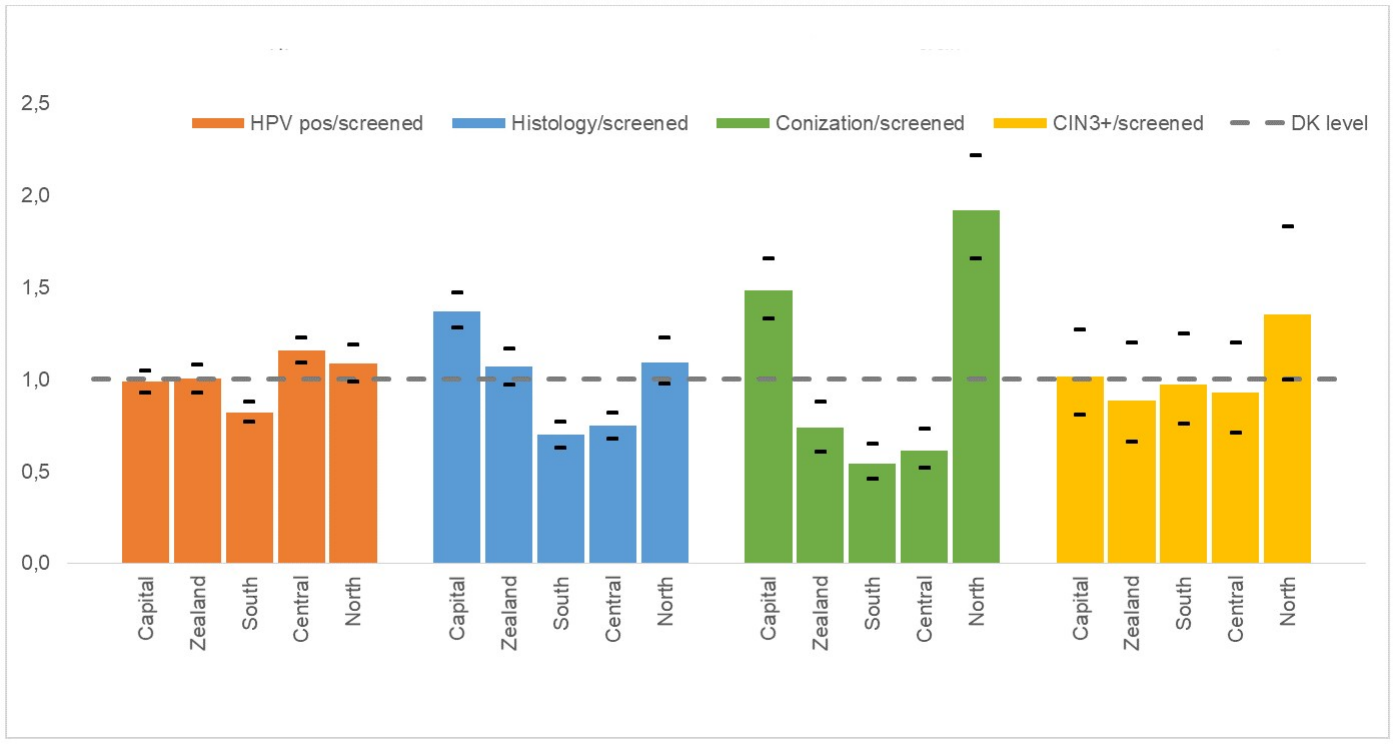
Regional proportions of women who were HPV positive, had histology performed, had CIN3+, or underwent conization, compared to national average.

As shown in Fig 2 and Table 3, quite different proportions of screened women underwent biopsy and/or conization in the five regions, in spite of smaller variations in HPV positivity rate and CIN3+ detection. Accordingly, the number of conizations per CIN3+ differed substantially between regions from 1.8 in Southern Denmark to 4.7 in the Capital Region, Table 4. The number of women with histology to CIN3+ cases diagnosed varied between regions, with Capital and Zealand regions above the national average. Capital Region had the highest numbers of both histology and conizations per CIN3+ case, whereas North Denmark Region had a high number of conization per CIN3+ case, while the number of histology per CIN3+ case was below the national average.

## Discussion

### Main findings

In 2017, 108,585 Danish women aged 69 years or older had cervical samples tested for HPV, and 4.1% were HPV-positive. By far the majority of the HPV-positive women had one or more follow-up tests taken, and about two-thirds had at least one histology result. Conization was performed in 1% of screened women, corresponding to 24% of HPV-positive women. Cervical cancer was detected in 37 women, and CIN3 in 300 women, which was a CIN3+ detection rate of 0.31% in screened women and of 7.5% in HPV-positive women. More than three women underwent conization per detected CIN3+ case. Proportions of screened women testing HPV-positive, undergoing histology or conization varied little below age 79 but were lower in women above this age. CIN3+ detection rates were similar across age.

While the CIN3+ detection rates varied little across the five Danish regions, there was a considerable variation in proportions of women undergoing histology and/or conization. As a consequence, there was approximately a two-fold variation in the numbers of biopsies/conizations per severe histology across regions.

### Other studies

A Swedish study including 1,051 women aged 60–89 years found that 4.1% were HPV- positive [12]. For HPV-positive women, testing was repeated after 3 months, and if negative again at 12 months. Overall, 30 (3%) women had two positive HPV-tests within one year, and 30% of those positive on first test were negative on both repeat-tests. In another Swedish study, where women initially HPV-negative were retested after a mean interval of 3.2 years, 2.6% were HPV-positive, of whom 52% were positive again on repeat-test after an average of 2.5 months [13]. In both studies, women with two positive HPV-tests had a high prevalence of CIN on histology, although mainly CIN1, but few had detectable cytological changes. Comparable results were obtained in another study where HPV-test was performed on self-collected samples [14]. When retesting HPV-positive or HPV-negative women, these studies thus demonstrated that test results in elderly women vary over even short time spans, although it is unknown to which extent this is due to cleared infections, reinfection, reactivation of latent HPV-infection or difficulty in obtaining representative samples from elderly women. In these Swedish studies, fewer severe histology lesions were found than in ours. This may be related to sample size, selection of studied women, to screening histories of studied women, or to the historically lower cervical cancer incidence in Sweden than in Denmark [15].

A Danish GRADE-based evidence review on management of elderly, HPV-positive women concluded that there is insufficient evidence on how to ensure reduction in cervical cancer risk while minimizing overtreatment [16]. Surveillance over time is complicated by the difficulty in obtaining representative samples from the cervix due to the age-related changes, and the Danish guidelines therefore recommend considering conization in women where the transformation zone cannot be visualized and/or biopsies have been unrepresentative.

The Horizon study compared screening by cytology to different HPV assays in Denmark [17]. For women aged 30–65 years screened with Cobas HPV test (which was also used in most regions for the one-time HPV screening of older women), the study found 129 biopsies and 37 CIN3+ cases among 465 women positive on Cobas, i.e. 8% of HPV positive women had CIN3+, and 3.5 biopsies were performed per CIN3+ case diagnosed.

A woman’s risk of cervical cancer in old age is related to her screening history [18,19], and at the population level, the cervical cancer incidence in a specific age-group depends on the screening history of the birth cohorts making up that age-group at that point in time [8]. The Danish data showed a steady decrease in HPV-prevalence after the age of 35 years [9]. 82% of cervical cancer cases diagnosed in women aged above 60 years occurred in never or insufficiently screened women, and the average age of non- or insufficiently screened cancer patients was 76 years, the same age as cancer incidence peaks in old age [9,20]. However, the Danish Pathology Register is only complete after 1998, and the screening history of older women is therefore underreported in the register. Consequently, studies show an increase in proportion screened over time, reflecting not only increased screening but also increased completeness of the pathology register [21].

A cohort study of more than 500,000 Swedish women found that screening between age 61 and 65 years was beneficial for women with an insufficient screening history, while there was no significant reduction in cervical cancer risk up to age 80 years in women adequately screened with normal test results at age 51–60 [18].

### Strengths and limitations

The availability of the national pathology register and unique identifiers ensured complete follow-up. However, data on deaths and emigrations were not available, and we were therefore unable to censor these women in the analysis. At the population level, we know that the birth cohorts targeted by the one-time HPV-screening were insufficiently screened, but individual data on the screening history, previous histology findings or therapeutic procedures were not available, as the pathology register data are incomplete before 1998.

For some women, conization was performed after a biopsy had revealed a severe lesion, but for other women the procedure was diagnostic, when a representative biopsy could not be obtained. For some women, a conization may have been performed even in case of a non-severe/no lesion to avoid lengthy follow-up with repeated cervical sampling. Some women may still be in follow-up with HPV test and/or biopsy and may still be conizised after the period covered in this study. Conization rates could thus be slightly higher than reported here, as a decision on end of follow-up is not coded.

Women whose HPV screening test was negative were not retested, so intermittent HPV-positivity, as in the Swedish studies referenced above, would not have been detected, and the prevalence of histological lesions in women testing HPV negative was unknown. This limits the ability of the study to fully estimate the cancer preventive effect of a single HPV test in these age groups.

Part of the variation between regions could theoretically stem from differences in age distribution. The aggregate RKKP data did not allow for age adjustment, but there is little difference across regions in the age distribution of women in the studied age-range [22]. We consider it unlikely that age specific participation in screening should vary across regions, and consequently we consider it unlikely that the observed regional differences in histology and conization rate derived from difference in age distributions.

### Clinical implications

The European guidelines for cervical cancer screening recommend that women exit cervical cancer screening at age 60–65 years provided they have had a recent negative test [23]. The US preventive services task force recommends against screening of women older than 65 years if they have had adequate prior screening and are not at high risk for cervical cancer [24].

A possible extension of existing screening programs beyond age 65 years has been subject of both scientific and political considerations in many countries. In Denmark, the decision was to target in incompletely screened birth cohorts with the one-time HPV screening described here. Our results suggest that, for these cohorts, HPV screening can potentially prevent some of the morbidity and mortality from cervical cancer, as severe lesions (CIN3+) were detected in 7.5% of HPV-positive women, including 37 women with cervical cancer. However, not all CIN3+ cases progress to cancer, and some of the 37 detected cervical cancer cases might not have become symptomatic in the women’s remaining lifetime. Whether this screening intervention translates into fewer cervical cancer cases and deaths will be visible only after a longer follow-up.

The results may not, however, be generalizable to birth cohorts after 1948 who have had a systematic screening offer throughout their adult life. It is also important to note that, although women older than 65 years constitute an increasing proportion of cervical cancer cases, the incidence in older women has declined over the past decades [15,25], reflecting the progressively lower risk in each birth cohort [8]. The age-specific mortality has also declined over time [15]. With primary HPV screening being implemented in many countries, the risk in elderly women may be even lower in the future.

An alternative to a general extension of screening age might be to consider more personalized approaches, factoring in each woman´s age, screening history, comorbidities, general health and expected remaining lifespan [26] but more evidence is needed to estimate effects of both general and personalized strategies. In Denmark, complete cervical cancer screening results are available from the Danish Pathology Register for birth cohorts aging in the future, thus enabling studies comparing cancer risk after screening age for women who did or did not meet exit criteria, including women whose exit test was an HPV test.

In all screening initiatives, it is important to consider the potential harms in addition to the benefits. Although conization is generally considered a minor procedure, the risk of bleeding and complications like perforations may be elevated in older women, and age-related anatomical changes may render the procedure more difficult. In addition, the therapeutic effect of conization is not well documented for women over 60 years [16]. On the other hand, obtaining representative biopsies in older women is not always possible and therefore conization may be necessary for providing a diagnosis and can be preferable to avoid lengthy follow-up with repeated cervical sampling [16]. As shown in this study, the choice of management strategy has an important impact on the number of biopsies/conizations per findings of CIN2+ or CIN3+, and conceivably also on the number of women recalled for repeated tests in the absence of conization. Although differences in study design do not allow for direct comparison with the Horizon study, the results do suggest that the yield of CIN3+ compared to the number having histology results is less favorable for older women, with 8.3 histologies per CIN3+ in our study vs. 3.5 in Horizon.

## Conclusion

To address the concern about the relatively high proportion of cervical cancer cases diagnosed beyond screening age, in 2017, a one-time HPV-screening initiative was offered to Danish women born before 1948. These birth cohorts had not been offered sufficient systematic screening earlier in life. In total, 4.1% of participating women tested HPV-positive, and out of those 7.5% had CIN3+ detected. While these proportions varied little across the five Danish regions, there were considerable regional differences in proportions of women undergoing biopsy and conization, reflecting differences in management strategies.
