# Supplementary material for: Histological outcomes in HPV-screened elderly women in Denmark
Source: PLoS One. 2021 Feb 11;16(2):e0246902. doi: 10.1371/journal.pone.0246902 (PMC7877658; doi:10.1371/journal.pone.0246902)
Supplement: Attachment — Submitted filename: Response to Reviewers.docx [file pone.0246902.s001.docx]

To the editors,

We are grateful for the review of our manuscript Histological outcomes in HPV-screened elderly women in Denmark.

We have made the requested changes to style and formatting as per point 1 in the Decision letter. In accordance with point 2 in the decision letter, the updated competing interests statement is included in our cover letter.

We have read with great interest the reviewer comments and revised the manuscript to reflect their inputs. Below are our replies to their individual points, inserted in the text:

Reviewer #1: This is an important work that provides an insight of how to manage cervical cancer screening in women that may have not had because of their age an adequate screening history. The authors provide a cohort and a regional analysis of HPV detection, precancer cases detected and invasive cervical cancer detected. The geographical variability is noted as well as the cohort effect where older women have lower burden of disease. The authors identify the strength and limitation of the study. They are caution in indicating that the impact of this additional effort will be seen in follow up data on incidence and mortality from cervical cancer.
I have one consideration is that the authors report that out of 359,763 invited women, only 108,585 (30%) were screened. Was there any attempt to re/contact these women?. Given the number of cases detected among those screened it seems reasonable to try to capture more women. I could not see a discussion on this issue.

*Reply: We thank this reviewer for the comments on our paper and for raising the question regarding renewed contact to non-participating women. The screening initiative was part of the MoH’s National plan for cancer from 2017 and only provided for contacting the women once. We have clarified this in the Materials and methods section of the revised manuscript.*

Reviewer #2: This is an excellent manuscript reporting precancer/cancer outcomes in a 'catch-up' HPV screening population of women born before 1948 in Denmark. In general the results are well-described and sound, though several questions or clarifications remain to support the author's conclusion that these results do not justify a recommendation to increase the screening exit age (which may be appropriate but is not yet justified with the reported data, though the opportunity seems to exist):

*Reply: we thank the reviewer for the comments. We did not intend to make firm recommendations for the current screening program but the reviewer comments helped us realize that our manuscript could give this impression. The birth cohorts born before 1948, targeted by the one-time screening initiative, were not systematically offered screening throughout their adult life and therefore have a higher cervical cancer risk than cohorts exiting the screening program now (cf. Lynge et al., Eur J Cancer, 2017). Our discussion aimed to stress this fact and we have made several revisions throughout the discussion to clarify.*

1. As a comparator, the authors should have the ability to examine the rate of CIN3+ at younger ages among screened and HPV positive women (recognizing HPV testing has been more recently introduced into the national screening program, many large studies have been done in Denmark that could serve as a benchmark for comparison). Is the yield of CIN3+ per biopsy/cone similar, higher, lower compared to younger women? This seems to be a critical comparison in making an argument for screening women up to age 65 but not after given the high PPV of an HPV positive test observed here. Note that >95% of older women would just need to provide a cervicovaginal sample in the case of 1-2 additional screens in the lifetime for quite a considerable yield of precancer. Agree, however, that this must be balanced against harms, life expectancy, and women's preferences.

*Reply: We thank the reviewer for suggesting this comparison which we had not considered. Data from the current screening program are not reported in a way that enables for direct comparison. We therefore cite in the revised manuscript the Danish Horizon study (Rebolj et al., PLoS ONE, 2016), which compared cytology to HPV primary screening among women aged 30-65. Although results are not directly comparable to ours due to different study designs, they do suggest that the yield of CIN3+ per histology performed is better for women under 65 than for our study cohorts.*

2. The authors state on page 16, lines 253-254 that screening history for individual women is not available - given that some of their references as well as many others from Denmark report screening history in women diagnosed from cancer, it is unclear why a similar methodology could not be used to assess this important information. This helps to understand residual risk at older ages in the context of different screening histories, which is critical for setting policy on exit ages.

*Reply: the Danish Pathology Register is only complete from 1998 onwards, and therefore we do not have complete screening histories for women in the age groups concerned by the study.*

3. While the authors are correct that in Denmark 80% of cervical cancer cases over age 60 years were insufficiently or never screened, it should be noted that insufficient screening in the elderly may reflect not having been screened recently because they exited screening ages. Doesn't this reflect the screening exit policy (a general response), and not a need to capture non-compliant screeners (a personalized, individual response)? This is again why it seems important to document how many women had a screening record to meet exiting criteria against the detected CIN3+ in this study (see Hammer et al. J Low Gen Tract Dis. 2018;22(1).). Linking screening history in this study would be broadly informative as this information is otherwise not available!

*Reply: We are grateful for suggesting this study by Hammer et al. to supplement the one already cited, and we now include both in the references. As discussed in the 2018 article by Hammer et al., the lack of screening history reported may be due to incompleteness of the register as well as to not being screened. Consequently the study shows an increase in proportion screened over time, reflecting not only increased screening but also increased completeness of the pathology register.*

*In all studies on cervical cancer screening and incidence, the effects of age, period and cohort are intertwined, rendering the discussion quite complicated. The reviewer comments helped us realize that some of our points may not have been sufficiently well explained and we have rephrased parts of the discussion.*

4. The authors conclude not to recommend extending screening exit ages due to the complications of diagnosis/treatment at older ages. Is there not an option to use the same registers to examine any adverse events that followed the procedures in these women and compare these to AEs in HPV-positive younger women? So far, these arguments are anecdotal and need to be substantiated with quantifiable data.

*Reply: The observations reported by clinicians who have the impression that complication rates are indeed increased merit attention because of potential impact on the harm vs. benefit of screening. We therefore strongly agree with the reviewer that there is a need for documentation and quantification. However, data on treatment and complications are not in the pathology register and studying these is beyond the scope of the present report on histology findings in the one-time screening.*

We have found the reviewer comments most helpful in improving the manuscript and look forward to renewed decision from the editor.

On behalf of the authors,

Yours sincerely,

Gry St-Martin
