# Supplementary material for: Histological outcomes in HPV-screened elderly women in Denmark
Source: PLoS One. 2021 Feb 11;16(2):e0246902. doi: 10.1371/journal.pone.0246902 (PMC7877658; doi:10.1371/journal.pone.0246902)
Supplement: Peer review history

# Peer review: Decision Letter 0

**DOI:** 10.1371/journal.pone.0246902.r001

**Authors:** Clement A. Adebamowo

26 Nov 2020

PONE-D-20-34114

Histological outcomes in HPV-screened elderly women in Denmark

PLOS ONE

Dear Dr. St-Martin,

Thank you for submitting your manuscript to PLOS ONE. After careful consideration, we feel that it has merit but does not fully meet PLOS ONE’s publication criteria as it currently stands. Therefore, we invite you to submit a revised version of the manuscript that addresses the points raised during the review process.

Please submit your revised manuscript by Jan 10 2021 11:59PM. If you will need more time than this to complete your revisions, please reply to this message or contact the journal office at plosone@plos.org. When you're ready to submit your revision, log on to https://www.editorialmanager.com/pone/ and select the 'Submissions Needing Revision' folder to locate your manuscript file.

Please include the following items when submitting your revised manuscript:

- A rebuttal letter that responds to each point raised by the academic editor and reviewer(s). You should upload this letter as a separate file labeled 'Response to Reviewers'.
- A marked-up copy of your manuscript that highlights changes made to the original version. You should upload this as a separate file labeled 'Revised Manuscript with Track Changes'.
- An unmarked version of your revised paper without tracked changes. You should upload this as a separate file labeled 'Manuscript'.

If you would like to make changes to your financial disclosure, please include your updated statement in your cover letter. Guidelines for resubmitting your figure files are available below the reviewer comments at the end of this letter.

If applicable, we recommend that you deposit your laboratory protocols in protocols.io to enhance the reproducibility of your results. Protocols.io assigns your protocol its own identifier (DOI) so that it can be cited independently in the future. For instructions see: http://journals.plos.org/plosone/s/submission-guidelines#loc-laboratory-protocols

We look forward to receiving your revised manuscript.

Kind regards,

Clement A. Adebamowo, BM, ChB Hons; FWACS, FACS, ScD, FASCO

Academic Editor

PLOS ONE

Journal Requirements:

When submitting your revision, we need you to address these additional requirements.

1. Please ensure that your manuscript meets PLOS ONE's style requirements, including those for file naming. The PLOS ONE style templates can be found at

https://journals.plos.org/plosone/s/file?id=wjVg/PLOSOne_formatting_sample_main_body.pdf and

https://journals.plos.org/plosone/s/file?id=ba62/PLOSOne_formatting_sample_title_authors_affiliations.pdf

2. Thank you for stating the following in the Competing Interests section:

"I have read the journal's policy and the authors of this manuscript have the following competing interests:

Dr. Andersen reports non-financial support from Roche and Axlab, outside the submitted work

Dr. Lynge reports non-financial support from Roche for other studies, during the conduct of this study.

The remaining authors have no conflicts of interest."

Please confirm that this does not alter your adherence to all PLOS ONE policies on sharing data and materials, by including the following statement: "This does not alter our adherence to  PLOS ONE policies on sharing data and materials.” (as detailed online in our guide for authors http://journals.plos.org/plosone/s/competing-interests).  If there are restrictions on sharing of data and/or materials, please state these. Please note that we cannot proceed with consideration of your article until this information has been declared.

Please include your updated Competing Interests statement in your cover letter; we will change the online submission form on your behalf.

Please know it is PLOS ONE policy for corresponding authors to declare, on behalf of all authors, all potential competing interests for the purposes of transparency. PLOS defines a competing interest as anything that interferes with, or could reasonably be perceived as interfering with, the full and objective presentation, peer review, editorial decision-making, or publication of research or non-research articles submitted to one of the journals. Competing interests can be financial or non-financial, professional, or personal. Competing interests can arise in relationship to an organization or another person. Please follow this link to our website for more details on competing interests: http://journals.plos.org/plosone/s/competing-interests

[Note: HTML markup is below. Please do not edit.]

Reviewers' comments:

Reviewer's Responses to Questions

**Comments to the Author**

1. Is the manuscript technically sound, and do the data support the conclusions?

The manuscript must describe a technically sound piece of scientific research with data that supports the conclusions. Experiments must have been conducted rigorously, with appropriate controls, replication, and sample sizes. The conclusions must be drawn appropriately based on the data presented. 

Reviewer #1: Yes

Reviewer #2: Yes

**********

2. Has the statistical analysis been performed appropriately and rigorously? 

Reviewer #1: Yes

Reviewer #2: Yes

**********

3. Have the authors made all data underlying the findings in their manuscript fully available?

The PLOS Data policy requires authors to make all data underlying the findings described in their manuscript fully available without restriction, with rare exception (please refer to the Data Availability Statement in the manuscript PDF file). The data should be provided as part of the manuscript or its supporting information, or deposited to a public repository. For example, in addition to summary statistics, the data points behind means, medians and variance measures should be available. If there are restrictions on publicly sharing data—e.g. participant privacy or use of data from a third party—those must be specified.

Reviewer #1: Yes

Reviewer #2: Yes

**********

4. Is the manuscript presented in an intelligible fashion and written in standard English?

PLOS ONE does not copyedit accepted manuscripts, so the language in submitted articles must be clear, correct, and unambiguous. Any typographical or grammatical errors should be corrected at revision, so please note any specific errors here.

Reviewer #1: Yes

Reviewer #2: Yes

**********

5. Review Comments to the Author

Please use the space provided to explain your answers to the questions above. You may also include additional comments for the author, including concerns about dual publication, research ethics, or publication ethics. (Please upload your review as an attachment if it exceeds 20,000 characters)

Reviewer #1: This is an important work that provides an insight of how to manage cervical cancer screening in women that may have not had because of their age an adequate screening history. The authors provide a cohort and a regional analysis of HPV detection, precancer cases detected and invasive cervical cancer detected. The geographical variability is noted as well as the cohort effect where older women have lower burden of disease. The authors identify the strength and limitation of the study. They are caution in indicating that the impact of this additional effort will be seen in follow up data on incidence and mortality from cervical cancer.

I have one consideration is that the authors report that out of 359,763 invited women, only 108,585 (30%) were screened. Was there any attempt to re/contact these women?. Given the number of cases detected among those screened it seems reasonable to try to capture more women. I could not see a discussion on this issue.

Reviewer #2: This is an excellent manuscript reporting precancer/cancer outcomes in a 'catch-up' HPV screening population of women born before 1948 in Denmark. In general the results are well-described and sound, though several questions or clarifications remain to support the author's conclusion that these results do not justify a recommendation to increase the screening exit age (which may be appropriate but is not yet justified with the reported data, though the opportunity seems to exist):

1. As a comparator, the authors should have the ability to examine the rate of CIN3+ at younger ages among screened and HPV positive women (recognizing HPV testing has been more recently introduced into the national screening program, many large studies have been done in Denmark that could serve as a benchmark for comparison). Is the yield of CIN3+ per biopsy/cone similar, higher, lower compared to younger women? This seems to be a critical comparison in making an argument for screening women up to age 65 but not after given the high PPV of an HPV positive test observed here. Note that >95% of older women would just need to provide a cervicovaginal sample in the case of 1-2 additional screens in the lifetime for quite a considerable yield of precancer. Agree, however, that this must be balanced against harms, life expectancy, and women's preferences.

2. The authors state on page 16, lines 253-254 that screening history for individual women is not available - given that some of their references as well as many others from Denmark report screening history in women diagnosed from cancer, it is unclear why a similar methodology could not be used to assess this important information. This helps to understand residual risk at older ages in the context of different screening histories, which is critical for setting policy on exit ages.

3. While the authors are correct that in Denmark 80% of cervical cancer cases over age 60 years were insufficiently or never screened, it should be noted that insufficient screening in the elderly may reflect not having been screened recently because they exited screening ages. Doesn't this reflect the screening exit policy (a general response), and not a need to capture non-compliant screeners (a personalized, individual response)? This is again why it seems important to document how many women had a screening record to meet exiting criteria against the detected CIN3+ in this study (see Hammer et al. J Low Gen Tract Dis. 2018;22(1).). Linking screening history in this study would be broadly informative as this information is otherwise not available!

4. The authors conclude not to recommend extending screening exit ages due to the complications of diagnosis/treatment at older ages. Is there not an option to use the same registers to examine any adverse events that followed the procedures in these women and compare these to AEs in HPV-positive younger women? So far, these arguments are anecdotal and need to be substantiated with quantifiable data.

**********

6. PLOS authors have the option to publish the peer review history of their article (what does this mean?). If published, this will include your full peer review and any attached files.

If you choose “no”, your identity will remain anonymous but your review may still be made public.

**Do you want your identity to be public for this peer review?** For information about this choice, including consent withdrawal, please see our Privacy Policy.

Reviewer #1: **Yes: **Silvia de Sanjose

Reviewer #2: No

[NOTE: If reviewer comments were submitted as an attachment file, they will be attached to this email and accessible via the submission site. Please log into your account, locate the manuscript record, and check for the action link "View Attachments". If this link does not appear, there are no attachment files.]

While revising your submission, please upload your figure files to the Preflight Analysis and Conversion Engine (PACE) digital diagnostic tool, https://pacev2.apexcovantage.com/. PACE helps ensure that figures meet PLOS requirements. To use PACE, you must first register as a user. Registration is free. Then, login and navigate to the UPLOAD tab, where you will find detailed instructions on how to use the tool. If you encounter any issues or have any questions when using PACE, please email PLOS at figures@plos.org. Please note that Supporting Information files do not need this step.

# Author comment: Author response to Decision Letter 0

**DOI:** 10.1371/journal.pone.0246902.r002

13 Jan 2021

To the editors,

We are grateful for the review of our manuscript Histological outcomes in HPV-screened elderly women in Denmark.

We have made the requested changes to style and formatting as per point 1 in the Decision letter. In accordance with point 2 in the decision letter, the updated competing interests statement is included in our cover letter. 

We have read with great interest the reviewer comments and revised the manuscript to reflect their inputs. Below are our replies to their individual points, inserted in the text:

Reviewer #1: This is an important work that provides an insight of how to manage cervical cancer screening in women that may have not had because of their age an adequate screening history. The authors provide a cohort and a regional analysis of HPV detection, precancer cases detected and invasive cervical cancer detected. The geographical variability is noted as well as the cohort effect where older women have lower burden of disease. The authors identify the strength and limitation of the study. They are caution in indicating that the impact of this additional effort will be seen in follow up data on incidence and mortality from cervical cancer.

I have one consideration is that the authors report that out of 359,763 invited women, only 108,585 (30%) were screened. Was there any attempt to re/contact these women?. Given the number of cases detected among those screened it seems reasonable to try to capture more women. I could not see a discussion on this issue.

Reply: We thank this reviewer for the comments on our paper and for raising the question regarding renewed contact to non-participating women. The screening initiative was part of the MoH’s National plan for cancer from 2017 and only provided for contacting the women once. We have clarified this in the Materials and methods section of the revised manuscript. 

Reviewer #2: This is an excellent manuscript reporting precancer/cancer outcomes in a 'catch-up' HPV screening population of women born before 1948 in Denmark. In general the results are well-described and sound, though several questions or clarifications remain to support the author's conclusion that these results do not justify a recommendation to increase the screening exit age (which may be appropriate but is not yet justified with the reported data, though the opportunity seems to exist):

Reply: we thank the reviewer for the comments. We did not intend to make firm recommendations for the current screening program but the reviewer comments helped us realize that our manuscript could give this impression. The birth cohorts born before 1948, targeted by the one-time screening initiative, were not systematically offered screening throughout their adult life and therefore have a higher cervical cancer risk than cohorts exiting the screening program now (cf. Lynge et al., Eur J Cancer, 2017). Our discussion aimed to stress this fact and we have made several revisions throughout the discussion to clarify.

1. As a comparator, the authors should have the ability to examine the rate of CIN3+ at younger ages among screened and HPV positive women (recognizing HPV testing has been more recently introduced into the national screening program, many large studies have been done in Denmark that could serve as a benchmark for comparison). Is the yield of CIN3+ per biopsy/cone similar, higher, lower compared to younger women? This seems to be a critical comparison in making an argument for screening women up to age 65 but not after given the high PPV of an HPV positive test observed here. Note that >95% of older women would just need to provide a cervicovaginal sample in the case of 1-2 additional screens in the lifetime for quite a considerable yield of precancer. Agree, however, that this must be balanced against harms, life expectancy, and women's preferences.

Reply: We thank the reviewer for suggesting this comparison which we had not considered. Data from the current screening program are not reported in a way that enables for direct comparison. We therefore cite in the revised manuscript the Danish Horizon study (Rebolj et al., PLoS ONE, 2016), which compared cytology to HPV primary screening among women aged 30-65. Although results are not directly comparable to ours due to different study designs, they do suggest that the yield of CIN3+ per histology performed is better for women under 65 than for our study cohorts.

2. The authors state on page 16, lines 253-254 that screening history for individual women is not available - given that some of their references as well as many others from Denmark report screening history in women diagnosed from cancer, it is unclear why a similar methodology could not be used to assess this important information. This helps to understand residual risk at older ages in the context of different screening histories, which is critical for setting policy on exit ages.

Reply: the Danish Pathology Register is only complete from 1998 onwards, and therefore we do not have complete screening histories for women in the age groups concerned by the study. 

3. While the authors are correct that in Denmark 80% of cervical cancer cases over age 60 years were insufficiently or never screened, it should be noted that insufficient screening in the elderly may reflect not having been screened recently because they exited screening ages. Doesn't this reflect the screening exit policy (a general response), and not a need to capture non-compliant screeners (a personalized, individual response)? This is again why it seems important to document how many women had a screening record to meet exiting criteria against the detected CIN3+ in this study (see Hammer et al. J Low Gen Tract Dis. 2018;22(1).). Linking screening history in this study would be broadly informative as this information is otherwise not available!

Reply: We are grateful for suggesting this study by Hammer et al. to supplement the one already cited, and we now include both in the references. As discussed in the 2018 article by Hammer et al., the lack of screening history reported may be due to incompleteness of the register as well as to not being screened. Consequently the study shows an increase in proportion screened over time, reflecting not only increased screening but also increased completeness of the pathology register.

In all studies on cervical cancer screening and incidence, the effects of age, period and cohort are intertwined, rendering the discussion quite complicated. The reviewer comments helped us realize that some of our points may not have been sufficiently well explained and we have rephrased parts of the discussion.

4. The authors conclude not to recommend extending screening exit ages due to the complications of diagnosis/treatment at older ages. Is there not an option to use the same registers to examine any adverse events that followed the procedures in these women and compare these to AEs in HPV-positive younger women? So far, these arguments are anecdotal and need to be substantiated with quantifiable data.

Reply: The observations reported by clinicians who have the impression that complication rates are indeed increased merit attention because of potential impact on the harm vs. benefit of screening. We therefore strongly agree with the reviewer that there is a need for documentation and quantification. However, data on treatment and complications are not in the pathology register and studying these is beyond the scope of the present report on histology findings in the one-time screening.

We have found the reviewer comments most helpful in improving the manuscript and look forward to renewed decision from the editor.

On behalf of the authors,

Yours sincerely,

Gry St-Martin

Attachment Submitted filename: Response to Reviewers.docx Click here for additional data file.

# Peer review: Decision Letter 1

**DOI:** 10.1371/journal.pone.0246902.r003

**Authors:** Clement A. Adebamowo

28 Jan 2021

Histological outcomes in HPV-screened elderly women in Denmark

PONE-D-20-34114R1

Dear Dr. St-Martin,

We’re pleased to inform you that your manuscript has been judged scientifically suitable for publication and will be formally accepted for publication once it meets all outstanding technical requirements.

Within one week, you’ll receive an e-mail detailing the required amendments. When these have been addressed, you’ll receive a formal acceptance letter and your manuscript will be scheduled for publication.

An invoice for payment will follow shortly after the formal acceptance. To ensure an efficient process, please log into Editorial Manager at http://www.editorialmanager.com/pone/, click the 'Update My Information' link at the top of the page, and double check that your user information is up-to-date. If you have any billing related questions, please contact our Author Billing department directly at authorbilling@plos.org.

If your institution or institutions have a press office, please notify them about your upcoming paper to help maximize its impact. If they’ll be preparing press materials, please inform our press team as soon as possible -- no later than 48 hours after receiving the formal acceptance. Your manuscript will remain under strict press embargo until 2 pm Eastern Time on the date of publication. For more information, please contact onepress@plos.org.

Kind regards,

Clement A. Adebamowo, BM, ChB Hons; FWACS, FACS, ScD, FASCO

Academic Editor

PLOS ONE

Additional Editor Comments (optional):

Reviewers' comments:

Reviewer's Responses to Questions

**Comments to the Author**

1. If the authors have adequately addressed your comments raised in a previous round of review and you feel that this manuscript is now acceptable for publication, you may indicate that here to bypass the “Comments to the Author” section, enter your conflict of interest statement in the “Confidential to Editor” section, and submit your "Accept" recommendation.

Reviewer #1: All comments have been addressed

Reviewer #2: All comments have been addressed

**********

2. Is the manuscript technically sound, and do the data support the conclusions?

The manuscript must describe a technically sound piece of scientific research with data that supports the conclusions. Experiments must have been conducted rigorously, with appropriate controls, replication, and sample sizes. The conclusions must be drawn appropriately based on the data presented. 

Reviewer #1: Partly

Reviewer #2: (No Response)

**********

3. Has the statistical analysis been performed appropriately and rigorously? 

Reviewer #1: Yes

Reviewer #2: (No Response)

**********

4. Have the authors made all data underlying the findings in their manuscript fully available?

The PLOS Data policy requires authors to make all data underlying the findings described in their manuscript fully available without restriction, with rare exception (please refer to the Data Availability Statement in the manuscript PDF file). The data should be provided as part of the manuscript or its supporting information, or deposited to a public repository. For example, in addition to summary statistics, the data points behind means, medians and variance measures should be available. If there are restrictions on publicly sharing data—e.g. participant privacy or use of data from a third party—those must be specified.

Reviewer #1: Yes

Reviewer #2: (No Response)

**********

5. Is the manuscript presented in an intelligible fashion and written in standard English?

PLOS ONE does not copyedit accepted manuscripts, so the language in submitted articles must be clear, correct, and unambiguous. Any typographical or grammatical errors should be corrected at revision, so please note any specific errors here.

Reviewer #1: Yes

Reviewer #2: (No Response)

**********

6. Review Comments to the Author

Please use the space provided to explain your answers to the questions above. You may also include additional comments for the author, including concerns about dual publication, research ethics, or publication ethics. (Please upload your review as an attachment if it exceeds 20,000 characters)

Reviewer #1: No additional comments. The authors have provided the review on the topics required. The flow of the paper is good and the data presented is very relevant for the management of older cohorts.

Reviewer #2: (No Response)

**********

7. PLOS authors have the option to publish the peer review history of their article (what does this mean?). If published, this will include your full peer review and any attached files.

If you choose “no”, your identity will remain anonymous but your review may still be made public.

**Do you want your identity to be public for this peer review?** For information about this choice, including consent withdrawal, please see our Privacy Policy.

Reviewer #1: No

Reviewer #2: No

# Editor report: Acceptance letter

**DOI:** 10.1371/journal.pone.0246902.r004

**Authors:** Clement A. Adebamowo

3 Feb 2021

PONE-D-20-34114R1 

Histological outcomes in HPV-screened elderly women in Denmark 

Dear Dr. St-Martin:

I'm pleased to inform you that your manuscript has been deemed suitable for publication in PLOS ONE. Congratulations! Your manuscript is now with our production department. 

If your institution or institutions have a press office, please let them know about your upcoming paper now to help maximize its impact. If they'll be preparing press materials, please inform our press team within the next 48 hours. Your manuscript will remain under strict press embargo until 2 pm Eastern Time on the date of publication. For more information please contact onepress@plos.org.

If we can help with anything else, please email us at plosone@plos.org. 

Thank you for submitting your work to PLOS ONE and supporting open access. 

Kind regards, 

PLOS ONE Editorial Office Staff

on behalf of

Dr. Clement A. Adebamowo 

Academic Editor

PLOS ONE
